# Colour-Blindness

**Published:** 1890-02-01

**Authors:** 


### The Doctor's Pulpit.

### COLOUR-BLINDNESS.

Four per cent. of the male population of this country are colour-blind. Such were the statistics given by Mr. Brudenell Carter at the Society of Arts last week. To most people the fact is a totally uninteresting one, for few have any need of an accurate colour-sense, and it may be that some of us are colour-blind without knowing it. Roughly speaking, to only two sets of people does it matter much whether or not their colour-sight is perfect —to sailors and railway workmen. These two are guided in their work by coloured lights, and that work is the safe conveyance of persons and property. Both may be endangered by colour-blindness.

It seems almost incredible to a person possessed of the normal colour-sense that anyone is unable to tell a red light from a green one. Do they both look the same to the colour-blind ? he asks. No. If they did it would be the easiest thing in the world to discover colour-blindness, and experience has shown that detection is often difficult. To a red-blind person—that is, one who is blind to red—the red lights appear a duller shade of green, something perhaps between green and grey, and he has learned that other people call that dull tint "red." If shown a red glass he would name it rightly, though he would see it wrong. But suppose that in a mist at sea or a snow-storm on land the green lamp showed a dull, blurred colour, he would think it was the tint he had been taught to call red, and act accordingly, perhaps with dangerous results. The converse would happen with a "green-blind" man. It is evident, then, that some test of normal vision should be applied to all who seek employment as engine-drivers, pilots, or any other post of similar responsibility.

In theory there is such a test. The railway men are asked to name the signals as they are successively held up, and, though merchant sailors are exempt from any test, the officers on board merchant vessels are examined by the Board of Trade before certificates are granted to them. But great dissatisfaction is felt at those examinations, and the most significant comment on them is that some shipowners refuse to accept them, and insist that all candidates for posts on board their vessels shall submit to another examination at the hands of an expert, who by no means invariably confirms the Board of Trade. One thing against the Board of Trade examination is that a candidate can go up as often as he likes. Now, colour-blindness is an incurable disease. A man who suffers from it at twenty-five has probably had it all his life, though it may be induced by some accident, and, however acquired, it can never be got rid of. But an intelligent man, after one or two failures, may be so "coached" as to pass the colour test correctly in the examination-room, though in actual practice he may fail entirely.

Again, though a candidate may go up for examination as often as he will, there is no compulsory re-examination. Yet any accidental injury may affect the eye and cause deterioration of the colour-sense. It is perhaps asking too much of human nature to expect that a man will offer himself for an examination which he suspects will result in his losing his situation. But it is therefore the more needful that the responsible authorities should hold regular re-examinations of those who hold their certificate. At least, men might be examined at the age of forty or forty-five, when the noon of life is reached, and constitutional changes begin. Perhaps it is as yet needless to insist on all sailors having perfect sight. Such a thing seems desirable ; but, after all, a common sailor is very rarely entrusted with the sole responsibility of a ship's course. The officer, whom the sailors must obey —and everyone knows what prompt and implicit obedience is required on board ship—is in a different position. Even though his subordinates know that "someone has blundered," the rule is that it is "theirs not to reason why" ; they are to do as they are told. Therefore, the man who tells them what to do should be thoroughly competent, as no colour-blind person can be. It is hard, bitterly hard, for a man to be rejected after, it may be, years of service without an accident. He might go on to the end of his days without any mishap occurring; but if such did occur, scores of lives might be sacrificed. In such a case the many must be regarded rather than the one.

What, then, is the best test for colour-blindness ? A very simple one—that known as Holmgren's wool test. Holmgren, a Swede, and a professor at the University of Upsala, was the first to direct public attention to the frequency with which colour-blindness is met with, and the risks implied in it. The test he has invented is so obvious, and when properly performed seems so satisfactory in its results, that the only cause for wonder is that it is not generally adopted. A number of skeins of wool are spread out on a white cloth in clear daylight, but not in direct sunshine. The colours are green of all shades, grey, drab, pink, and yellow. The candidate is given a skein of pale green wool, and asked to pick out the other skeins of the same colour without distinction of shade. Ninety-six per cent. will go through the test with ease ; but the remaining four will probably match the green with some of the other tints, the "confusion colours" as they are aptly called. This is the first test ; in the second the test colour is a reddish purple, a combination of red and violet. To the green-blind this combination seems grey, and he selects the skeins of that colour. The red-blind, on the other hand, does not see the red tint in the purple at all, and he matches it with blue or violet. The third test is to match a skein of bright red. The red-blind sets with this dark green or dark brown ; the green-blind matches it with brighter shades of these colours. The first test tells if colour-blindness exists ; the second shows whether it is red or green that has no existence for the candidate ; the third confirms the deductions drawn from the other two.

Where complete colour-blindness exists, a decision is comparatively easy to come to if Holmgren's test is carried out with due regard to the conditions he lays down. But, as in other defects of vision, there are many degrees between complete colour-blindness and normal sight. Such a thing as a feeble colour-sense is difficult to discover, and still more difficult to decide upon with due regard to the requirements of practical life. It would be interesting to know how many of us have a real colour-sense—a genuine enjoyment of clear, pure tints. Ruskin places the appreciation of colour high among the requirements of healthy national life. This seems fantastic, but is it so, indeed ? Does not a true—not a morbid or exaggerated—sensitiveness to the hues of dawn or sunset, the varied tints of the budding or fading leaves the colours of flowers, animals, stones, show a certain health of mind and body not too easily attainable in this generation of ours. In our great cities, with their smoke-dimmed skies and their soot-stained rivers, is a real colour-sense—something more than just the power to distinguish red from green—even possibly attainable ? Only to those who frequently refresh themselves with a sight of nature as God made it. Townsfolk are often to a certain degree colour-blind. The grey street, the grey houses, the grey sky, do not depress them; they do not feel the absence of colour in their surroundings. Sometimes this is accompanied by a certain mental colour-blindness, an incapacity for seeing the humour and pathos, the variety and charm of human life. Everything is dull to people thus afflicted, nearly everything is dreary. It is such people who care most for newspaper records of atrocious crimes, who like sensational improbabilities in drama and novel, because their dull sense is blind to the more delicate shades of real life. Whether this is not sometimes accompanied by a general blunting of the moral sense to the commands of simple daily duty is a point which might be worth enquiring into. Mr. Brudenell Carter would deserve even better of the community than he does if he could elucidate it.

